# Surgical Treatment of Multiple Bone Cysts Using a Platelet-Rich Fibrin and BoneAlbumin Composite Graft: A Case Report

**DOI:** 10.3390/reports7010007

**Published:** 2024-01-22

**Authors:** Martin Major, Márton Kivovics, Bence Tamás Szabó, Tamás Déri, Melinda Polyák, Noémi Piroska Jákob, Dániel Csete, Attila Mócsai, Zsolt Németh, György Szabó

**Affiliations:** 1 Department of Oro-Maxillofacial Surgery and Stomatology, Semmelweis University, 1085 Budapest, Hungary; nemeth.zsolt@semmelweis.hu; 2 Department of Community Dentistry, Semmelweis University, 1085 Budapest, Hungary; 3 Department of Oral Diagnostics, Semmelweis University, 1085 Budapest, Hungary; szabo.bence.tamas@semmelweis.hu; 4 Department of Prosthodontics, Semmelweis University, 1085 Budapest, Hungary; 5 Department of Restorative Dentistry and Endodontics, Semmelweis University, 1085 Budapest, Hungary; polyak.melinda9409@gmail.com; 6 Department of Pathology and Experimental Cancer Research, Semmelweis University, 1085 Budapest, Hungary; 7 Department of Physiology, Faculty of Medicine, Semmelweis University, 1085 Budapest, Hungarymocsai.attila@semmelweis.hu (A.M.)

**Keywords:** Gorlin–Goltz syndrome, keratocyst, PRF, BoneAlbumin, implantation, case report

## Abstract

Promising research results have been obtained on the tissue-regeneration properties of PRF (platelet-rich fibrin) in dentistry and maxillofacial surgery. PRF presumably promotes healing and accelerates ossification. In this case report, the patient had a history of Gorlin–Goltz syndrome, also called nevoid basal cell carcinoma syndrome, an autosomal dominant neurocutaneous disease that was known for many years. As a consequence, cysts were detected in both the mandible and maxilla. We performed decompression on this 37-year-old patient, followed by a cystectomy on an extensive lesion in the right angle of the mandible. One cyst from each side of the body mandible and one from the maxilla were completely enucleated, as determined using an intraoral exploration. The resulting bone defect was filled with a composite graft composed of a mixture of A-PRF and a serum albumin-coated bone allograft (BoneAlbumin). The wound was then covered with a PRF membrane. The surgical sites were closed per primam. The postoperative period was uneventful. Biopsies were performed after three and six months of healing for histological micromorphometry analyses. Dental implants were placed at the sampling site. Three months after the implantation, the ossified implants were fitted with superstructures. To date, no complications have appeared with the bone augmentation. The authors interpret from the findings that the combination of A-PRF and BoneAlbumin can be validated as a prosperous bone substitute. It can be safely implanted after a 3-month ossification period.

## 1. Introduction

In the fields of dentoalveolar surgery, maxillofacial surgery, and periodontology, it is often necessary to fill bone gaps with bone substitutes during the surgical treatment of cysts, impacted teeth, benign tumors, periodontal bone loss, etc. [1,2]. Odontogenic cysts can develop at any age. The location of the cyst primarily depends on its origin within the upper or lower jaw. Cysts can manifest as one or multiple cavities that are lined with a distinct cyst epithelium [3]. Asymmetric growth is characteristic, often leading to extreme size and significant bone loss as a consequence [4]. The most common types of cysts are radicular cysts, follicular cysts, and odontogenic keratocysts [5].

The complete removal of these cysts is crucial to avoid their recurrence, and therapy is significantly influenced by the location and extent of the lesion. Primary enucleation, which involves the complete removal of the cyst, may be considered as a potential treatment option. The decompression technique can be used to establish communication between the cyst and the oral cavity, resulting in a reduction in the size of the cyst. Then, another surgical procedure is necessary to remove the cyst wall when the cyst has successfully constricted [6,7]. The intralesional treatment of keratocystic odontogenic tumors can be complemented with Carnoy’s solution and cryotherapy with liquid nitrogen as adjunctive therapy [8]. The resulting cavity can be left untreated after enucleation, awaiting spontaneous bone regeneration from the formed blood clot or the adjacent bone walls covered with soft tissue [9]. The alternative is to fill the cavities with either autologous bone or bone-substitute material. Various bone-substitute materials have been described as potential fillers in such cases [10], but there is not enough evidence to support one treatment over the other or to determine which filling materials should be used. According to the literature, bone grafting can be a good solution to help complete healing if the defect length is greater than 1–2 cm and more than 50% of the bone volume is lost [11].

Platelet-rich fibrin (PRF) has been studied for its potential to aid in the healing of bone defects and soft tissue. Studies have investigated the use of PRF alone or in combination with other bone substitutes [12]. Platelet-rich fibrin (PRF) belongs to a new generation of platelet concentrates with simplified processing and without biochemical blood handling. There are different processing centrifugation protocols, such as the protocol for standard platelet-rich fibrin (S-PRF) (2700 rpm, 12 min) and advanced platelet-rich fibrin (A-PRF) (1500 rpm, 14 min). Due to the shorter centrifugation time, A-PRF has a more porous fibrin matrix and contains a higher concentration of leukocytes than the homogeneously produced PRF [13]. I-PRF (Injectable-PRF) is a liquid form of PRF that can be injected in a similar way to PRP, with the positive biological properties of PRF. It is centrifuged at 700 rpm for 3 min [12]. During the processing of PRF, the activation of platelets results in extensive degranulation and a significant release of cytokines, which, physiologically, can stimulate cell migration and proliferation within the fibrin matrix, initiating the healing process. The fundamental role in this initial mechanism is played by the three primary platelet cytokines due to their ability to promote cell migration and proliferation as well as induce fibrin matrix remodeling. Transforming growth factor β1 (TGF-β1) is the most potent cytokine fibrosis inducer and stimulates the significant production of matrix molecules such as collagen I and fibronectin by osteoblasts and fibroblasts [14]. PDGFs (platelet-derived growth factors) play a critical role in regulating the migration, proliferation, and survival of mesenchymal cell lineages [15,16]. Insulin-like growth factors (IGFs) I and II positively regulate the proliferation and differentiation of most cell types. Platelet degranulation results in the release of IGFs, which are initially present in high concentrations in the circulation [17].

Albumin, a major protein component in plasma [18], as a blood-derived product, has the potential for use in both autologous and allogeneic tissue engineering. It has proved to be successful in coating, scaffold construction, and hydrogel production, according to various reports. Ongoing advances suggest that albumin could be used in new regenerative medical products due to the abundant scientific proof of its regenerative traits and excellent biocompatibility [19].

In this manuscript, we present a case report of a 37-year-old male patient with a history of Gorlin–Goltz syndrome. Gorlin–Goltz syndrome is an autosomal dominant inherited condition that predisposes individuals to multiple types of cancers. Evident features include basal cell carcinoma, odontogenic keratocysts, calcification of the falx cerebri, bifid ribs, and pits on the palms and soles, and hypertelorism [20]. As a consequence, cysts were detected in both the mandible and maxilla. We used the decompression technique in the right ramus as well as primary enucleations with bone substitutions for the treatment of the cysts, depending on the location and the size of the lesions [21]. Biopsies and dental implantations were performed after the surgical sites had healed [22]. After three months of ossification, the stability of the implants was investigated using an Osstell device. The implants were then fitted with superstructures.

## 2. Detailed Case Description

The 37-year-old male patient who visited the Department of Oro-Maxillofacial Surgery and Stomatology at the Semmelweis University Faculty of Dentistry had a history of Gorlin–Goltz syndrome, also known as nevoid basal cell carcinoma syndrome. This case study was conducted in compliance with the ethical principles established in the Declaration of Helsinki. The patient was provided with comprehensive written information regarding the treatment objectives, procedures, potential hazards, and benefits before the commencement of any study procedures. Informed consent was acquired from the patient, with a clear explanation that participation was purely voluntary and that the patient had the autonomy to retract their consent at any time without any adverse consequences. This case report follows the instructions of the CARE guidelines [23].

**Figure 1:**
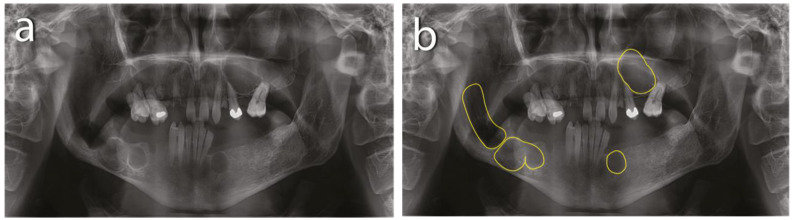
Preoperative X-rays: (**a**) residual roots and multiple keratocysts are visible on the orthopantomogram; (**b**) keratocysts are marked with yellow lines.

In this patient, multiple cystic lesions were accidentally detected in both the mandible and the maxilla through dental X-rays (Figure 1).

**Figure 2:**
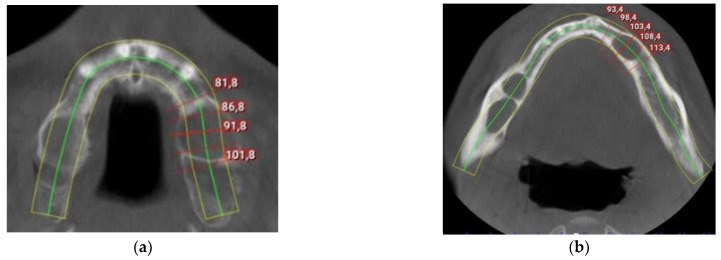
Preoperative CBCT scan: (**a**) maxillar keratocyst in the left upper region; (**b**) keratocysts in the mandible.

The intraoral examination revealed poor oral hygiene, with periodontitis, several missing teeth, and a few persistent roots. No visible manifestations of the cystic lesions in the oral cavity were detected. Panoramic radiographs and a CBCT scan revealed four cysts in both the mandible and maxilla (Figure 2). The first extensive radiolucent lesion spanned the right mandible from the condyle to the angle. A second, two-chamber osteolytic lesion was located in the right mandibular body in the molar area. Diagnostic imaging also revealed two cystic lesions in the left mandibular body in the premolar area and another in the maxilla, which was located between teeth 24 and 26 and was pushing their roots away from each other. The preparation for surgery consisted of scaling, a periodontic treatment, and dental sanation.

Due to the extensive nature of the first lesion in the right ramus mandible, decompression was considered to be an appropriate treatment plan for the lesion. Bone windows were created by excising the mucoperiosteum along with the bone in the retromolar region of the right mandibular buccal vestibule and crestal to the alveolar ridge. The decompression technique was used to produce a small window in the bone wall of the cyst in the retromolar region. A cyst-wall specimen was collected for a histological examination, confirming the diagnosis of a parakeratinized OKC. The cystic contents were evacuated, and the cystic cavity was packed with tape gauze soaked in 2% povidone–iodine for 3 days. A decompression draining device was constructed by a dental technician to ensure the cyst remained open. The device was equipped with a silicone tube that extends deeply into the cyst cavity, thereby reducing pressure and draining the contents of the cyst. The silicone tube is connected to an acrylic body, which, in our case, was attached to the lower teeth. The patient could remove this device himself to clean it twice a day; it was then re-placed into the cavity of the lesion. Then, a decision was made to completely evacuate the cyst, which was based on the pathology reports. After 10 months, a progressive reduction in radiolucency was noted, and a cystectomy was performed. The lesion was resected along with a disease-free bone margin. A histological examination confirmed the initial diagnosis of an OKC, a cyst with a connective tissue wall lined with a parakeratinized stratified squamous epithelium.

Primary enucleation could be performed in the case of all three remaining cysts due to their smaller size and better anatomical position. The same procedures were implemented under local anesthesia (Ultracain DS Forte, Sanofi-Aventis, Paris, France). Lingual paracrestal incisions and buccal double-releasing incisions were used on the mandible. Palatinal paracrestal incisions and buccal double-releasing incisions were used on the maxilla. The mucoperiosteal flap was then reflected to expose the soft cystic tissue. The complete enucleation of the cysts was achieved with a disease-free bone margin. The residual post-cystic cavities were augmented with a composite graft of a serum albumin-coated bone allograft (BoneAlbumin) and A-PRF. At the start of the operation, 40 mL of venous blood was collected from the patient without an anticoagulant for the preparation of A-PRF using Choukroun’s method [13]. Four tubes (A-PRF + tube, Process for PRF, Nice, France) were centrifuged for 14 min at 1300× *g* rpm (Duo Quattro Centrifuge, Process for PRF, Nice, France). After centrifugation, the fibrin clots were extracted from the tubes, placed in a metal box (PRF Box, Process for PRF, Nice, France), and pressed to remove the liquid content to obtain the membranes. BoneAlbumin (1.5–2 cm^3^) (BoneAlbumin, Orthosera Dental Zrt, Győr, Hungary) was mixed with fragments of the A-PRF membrane and the plasma that was squeezed from the metal box during the membrane preparation. The resulting bone graft was then delicately packed into the post-cystic cavities. The surgical lateral bone window was then covered using an A-PRF membrane. Finally, the mucoperiosteal flap was closed with single, interrupted, non-resorbable sutures. Antibiotics (1 g amoxicillin–clavulanic acid twice a day for seven days), anti-inflammatory medication (50 mg diclofenac three times a day for three days), and a chlorhexidine mouthwash (twice a day for seven days) were prescribed following the procedure. The sutures were removed after seven days, and no prostheses were used during the healing period. CBCT scans were obtained from all surgical sites after 3 and 6 months of healing prior to the implant placement. For the left mandibular cyst, the healing time was 6 months before biopsy sampling and implantation. For the right mandibular and maxillary cysts, biopsies were taken, and the implants were placed after 3 months of healing.

**Figure 3:**
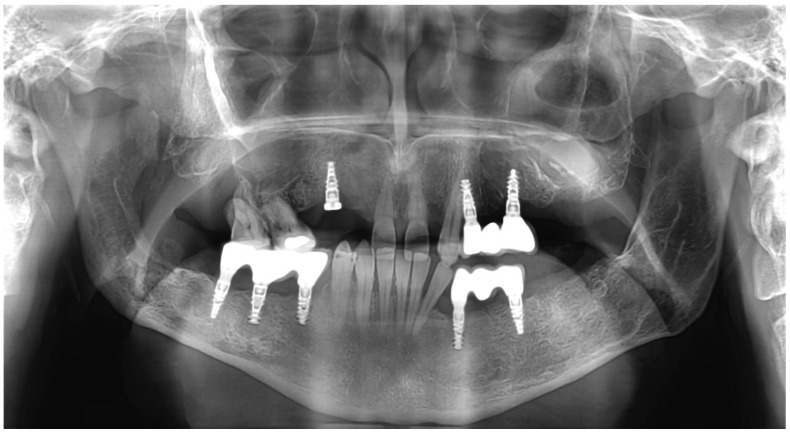
Postoperative picture of dentures and right maxillary region prosthetic care in progress.

After the necessary healing time, 8 implants were placed, following the same protocol. The implant procedure was performed using local anesthesia (Ultracain DS Forte, Sanofi-Aventis, Paris, France). A mid-crestal incision was used, and a full-thickness flap was raised. A modular trephine drill (Full-Tech Kft, Szigetszentmiklós, Hungary)—specifically designed for this study with an internal diameter of 2.5 mm, outer diameter of 3.25 mm, and length of 10 mm—was employed to initially collect the bone-core biopsy samples. The manufacturer’s drill was then used according to their protocol to continue the implant site preparation, and the implants were then immediately placed (SGS P5D implants with SBCT surface, SGS Dental, Budapest, Hungary). A two-stage healing protocol was employed, and non-resorbable sutures were used to close the flap. Antibiotics (1 g amoxicillin–clavulanic acid twice daily for seven days), anti-inflammatory medication (50 mg diclofenac three times a day for three days), and a chlorhexidine mouthwash (twice a day for seven days) were prescribed. The sutures were removed seven days after the implant placement. After three months of ossification, the implant stability quotient (ISQ) was measured using SmartPegs and an RFA device (Osstell IDx, Osstell AB, Göteborg, Sweden). The implants were then fitted with superstructures and dentures. A total of eight implants were placed; 4 were placed in the augmented area, and 4 were placed in the native bone (Figure 3).

**Figure 4:**
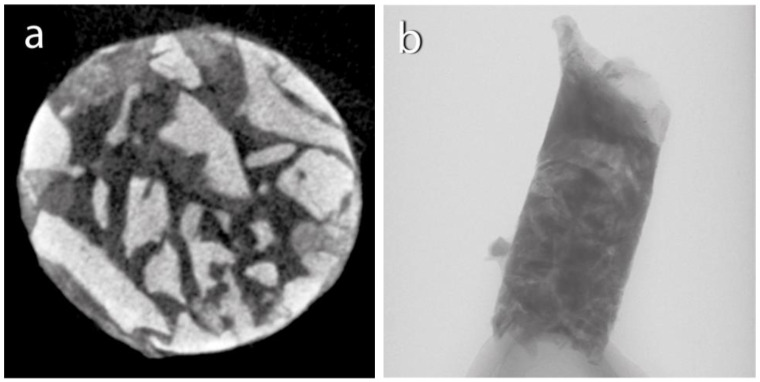
Micro-CT scan: (**a**) horizontal section; (**b**) vertical section.

**Figure 5:**
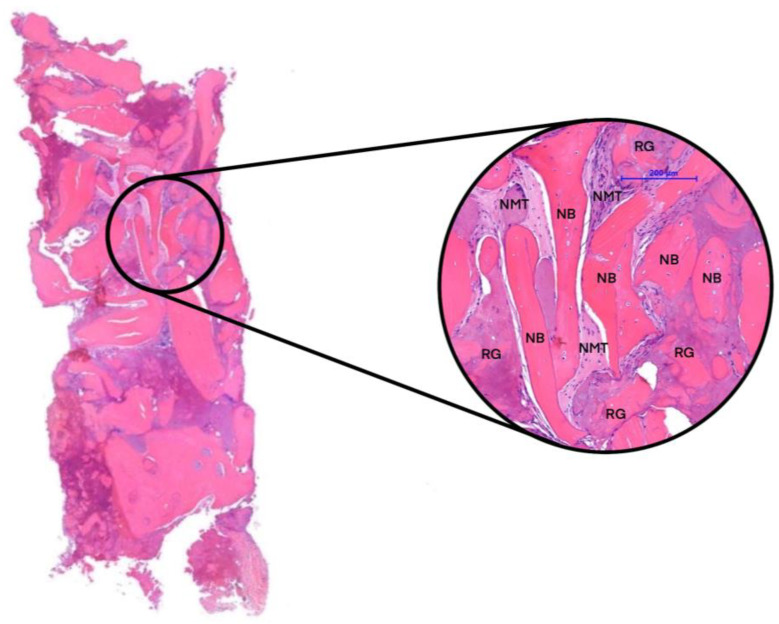
Histological section. Newly formed bone (NFB), residual graft particles (RGs), and non-mineralized tissue (NMT).

After the biopsy, the bone-core biopsy samples were scanned using a microcomputed tomography (µCT) scanner (Bruker 1272 X-ray microtomograph, Bruker µCT, Kontich, Belgium) (Figure 4). Following µCT scanning, the bone-core biopsy samples were histologically processed, and a histomorphometric analysis was conducted by a blinded examiner (Figure 5). The analysis investigated the samples taken after 6 months of healing and the samples taken after 3 months of healing. We examined the micromorphometric structure of the samples and, histologically, the amount of newly formed bone (NFB) and residual graft particles (RGs). The definitions of the pertinent morphometric parameters for the investigation are presented in Table 1.

## 3. Results

In our case study, biopsies were obtained from each implantation. The samples were scanned using µCT, and a micromorphometric analysis was performed. The samples included the augmented area after 3 months of healing, the augmented area after 6 months of healing, and native bone. A detailed analysis of the micromorphometric data is presented in Table 2 [24,25]. After undergoing µCT scanning, the bone-core biopsy samples proceeded to histological processing. The histological analysis detected signs of gradual graft resorption and remodeling in both groups within the augmented areas without any foreign-body reaction or inflammation. The percentages of newly formed bone (NFB), residual graft particles (RGs), and non-mineralized tissue (NMT) were calculated via manual segmentation. Native bone (NB) was excluded from the analysis for the samples obtained from the augmented area. Results of the histomorphometric analysis are presented in Table 3.

## 4. Discussion

The aims of this paper were to demonstrate the treatment options of a patient with multiple OKCs and a history of Gorlin–Goltz syndrome and to investigate the bone remodeling potential of a composite graft of BoneAlbumin and A-PRF after the primary enucleation of cysts and the filling of bone cavities. Micromorphometric and histomorphometric analyses were used to evaluate the characteristics of the augmented areas. The results of the present case report suggest that the combination of BoneAlbumin and A-PRF sticky bone is a suitable biomaterial for bone augmentation in the case of simultaneous cystectomies. The results of 3 months of healing were almost the same as those after the conventionally used 6-month healing period. This facilitated implant placement after a shorter healing time.

The total removal of these cysts remains the primary method to prevent their recurrence. The resulting bone defect can be left untreated, pending spontaneous bone regeneration, or filled with autologous bone or bone-substitute material [26]. Bone defects in the jaw, which are often associated with odontogenic cysts, have unique healing dynamics due to the continuous processes of bone resorption and apposition. The regeneration of bone is directly impacted by the size of the bone cavity, anatomical location, patient age, and other factors, with defect size being a significant factor in overall consolidation. A critical size defect (CSD), initially described by Schmitz et al., is a type of defect that does not completely and spontaneously recover, no matter how long it is observed [27]. It has been hypothesized that complete bone regeneration is not possible for cystic lesions with a diameter larger than CSDs that have been left without grafting. Schlegel et al. demonstrated that monocortical unfilled defects with a size of 10 × 10 mm presented incomplete bone regeneration after 52 weeks in an experimental animal study [28].

For osseous reconstruction, the gold standard in bone transplantation is an autogenous bone graft obtained from donor sites within or outside the mouth. These are predominantly implemented to regenerate bone imperfections located in the craniofacial area [29]. To circumvent the issue of donor morbidity arising from autogenous grafts, bone substitutes can be employed [30,31]. An ideal bone graft should possess osteoconductive, osteoinductive, and osteogenic properties. These criteria are only fulfilled by the gold standard, which is an autograft [32], although donor-site morbidity limits its use [33]. There are many studies in the literature where different bone substitutes have been presented as alternatives, each with a compromised healing potential compared with autografts [34,35].

Vivekanand et al. conducted research into various bone substitutions in cystic bone defects. They compared bovine-derived hydroxyapatite (BHA) and synthetic hydroxyapatite (SHA) graft materials as substitutes for bone grafts. The study concluded that both BHA and SHA graft materials were biocompatible to fill bone defects, resulting in less resorption and improved bone formation with equal effectiveness [36].

Serum albumin has been identified as an endogenous protein that plays an integral role in early bone regeneration. Horvathy and colleagues researched stem-cell attachment to serum albumin-coated demineralized bone matrices in a rat calvaria defect model. A bone defect was successfully treated in the experiment. Grafting with the use of BoneAlbumin resulted in a complete union and the formation of biomechanically functional new bone [37,38].

Kivovics et al. hypothesized that albumin-coated allografts could enhance bone remodeling compared with anorganic xenografts. Their study’s histological, histomorphometrical, and μCT analyses demonstrated that anorganic bovine bone mineral was incorporated into fresh bone, whereas BoneAlbumin chiefly underwent remodeling [39].

Simonffy et al. also investigated the healing effects of different bone substitutes, including BoneAlbumin. The goal of this study was to compare bovine xenograft (Bio-Oss) and BoneAlbumin in mandibular third molar extraction sockets bone grafting and to monitor bone remodeling and complications. The findings demonstrated that the use of BoneAlbumin in filling extraction sockets resulted in a higher level of bone regeneration compared with native bone buildup, a xenograft application, or the natural process of socket-healing without bone grafting [1].

Research in the literature has revealed that a serum albumin-coated bone allograft (BoneAlbumin) is an effective bone substitute, and not only in maxillofacial surgery. Gyulay et al. demonstrated the efficacy of BoneAlbumin in orthopedic surgery with a 7-year follow-up [40].

Opinions are divided in academic circles regarding the efficiency of platelet concentrates in enhancing wound healing and tissue regeneration [41,42]. Platelet-rich fibrin (PRF) was first introduced by Choukroun et al. in 2006 to enhance new bone formation in allografts and decrease the healing time to four months after maxillary sinus augmentation. The use of PRF presents a potential to boost the osteoinductive capabilities lost in bone-substitute fabrication by releasing autologous growth factors into the environment to promote faster remodeling [43,44]. The use of a low-speed centrifugation protocol to obtain A-PRF enhanced the quantity of growth factors in the scaffold and prolonged the shelf life by 14 days, thereby benefiting guided bone regeneration [45].

Other researchers have also documented their findings on the use of A-PRF and BoneAlbumin in their studies. Trimmel et al. investigated the microarchitecture of augmented bone following maxillary sinus augmentation using a composite graft of BoneAlbumin and A-PRF after healing periods of 3 months (test) and 6 months (control). Based on the results of the study, the employment of A-PRF and BoneAlbumin for two-stage maxillary sinus augmentation decreased the entire treatment duration by 3 months [31].

## 5. Conclusions

Based on our experience, the use of A-PRF and BoneAlbumin composite grafts can be a possible treatment option for the surgical treatment of multiple bone cysts. It is important to consider the anatomical location, clinical extent, size, age, and follow-up when making treatment decisions in each case. In our case, the use of PRF allowed us to safely perform the implantation after 3 months of healing. However, it is not possible to draw conclusions from a single case presentation, and much more research is needed to explore the effects of PRF on bone regeneration.

## Figures and Tables

**Table 1:** Definitions of the relevant morphometric variables.

Abbreviation	Variable	Description	Standard Unit
**BV/TV**	Bone volume fraction	Ratio of the segmented bone volume to the total volume of the region of interest	%
**BS/TV**	Bone surface density	Ratio of the segmented bone surface to the total volume of the region of interest	mm^−1^
**BS/BV**	Specific bone surface	Ratio of the segmented bone surface to the segmented bone volume	mm^−1^
**Tb.Th**	Trabecular thickness	Mean thickness of trabeculae assessed using direct 3D methods	mm
**Tb.Sp**	Trabecular separation	Mean distance between trabeculae assessed using direct 3D methods	mm
**Tb.Pf**	Trabecular bone-pattern factor	Index of connectivity of trabecular bone that calculates an index of the relative convexity or concavity of the total bone surface on the principle that concavity indicates connectivity (and the presence of ‘nodes’) and convexity indicates isolated disconnected structures (struts)	1/mm
**DA**	Degree of anisotropy	DA is 1 ¼ isotropic and >1 ¼ anisotropic by definition; DA is ¼ the length of the longest divided by the shortest mean intercept length vector	None
**SMI**	Structure model index	An indicator of the structure of trabeculae; SMI is 0 for parallel plates and 3 for cylindrical rods	None
**Po(op)**	Open porosity	Percent open porosity is the volume of open pores as a percent of the total VOI volume	%
**Po(tot)**	Total porosity	Total porosity is the volume of all open plus closed pores as a percent of the total volume of the region of interest	%
**Conn**	Connectivity	An effective and efficient method to compute the Euler connectivity in three dimensions is the Conneulor. This algorithm gauges a property termed ‘redundant connectivity’, which is the extent to which different parts of the object are interconnected. It quantifies the maximum number of connections in a structure that can be cut before the entire structure splits into two disjointed segments	None

**Table 2:** Results of the micromorphometric analysis.

Abbreviation	Sample	*n*	Mean
**BV/TV**	3 months of healing	2	25.7886
	6 months of healing	2	39.7210
	Native bone	4	41.0630
**BS/BV**	3 months of healing	2	0.0317
	6 months of healing	2	0.018.6
	Native bone	4	0.0146
**BS/TV**	3 months of healing	2	0.0069
	6 months of healing	2	0.0067
	Native bone	4	0.0059
**Tb.Th**	3 months of healing	2	151.8090
	6 months of healing	2	210.2448
	Native bone	4	216.2362
**Tb.Sp**	3 months of healing	2	316.6615
	6 months of healing	2	300.3770
	Native bone	4	256.2631
**Tb.Pf**	3 months of healing	2	0.0080
	6 months of healing	2	0.0055
	Native bone	4	0.0017
**DA**	3 months of healing	2	1.1206
	6 months of healing	2	1.1882
	Native bone	4	1.5026
**SMI**	3 months of healing	2	1.4071
	6 months of healing	2	1.9613
	Native bone	4	0.7452
**Po(tot)**	3 months of healing	2	74.2112
	6 months of healing	2	60.2789
	Native bone	4	58.9067
**Po(op)**	3 months of healing	2	74.2022
	6 months of healing	2	60.2427
	Native bone	4	58.9067
**Conn**	3 months of healing	2	1641.5
	6 months of healing	2	2160.5
	Native bone	4	1131.4

**Table 3:** Results of the histomorphometric analysis. The percentages of newly formed bone (NFB), residual graft particles (RGs), and non-mineralized tissue (NMT) were determined based on manual segmentation.

Groups	*n*	NFB	NMT	RG
**3 months of healing**	2	40.59%	30.82%	28.59%
**6 months of healing**	2	55.92%	27.95%	16.13%
**Native bone (NB)**	4	60.78%	39.22%	-

## Data Availability

The data presented in this study are available on request from the corresponding author. The data are not publicly available due to privacy.
